# A Naturally Occurring Defective DNA Satellite Associated with a Monopartite Begomovirus: Evidence for Recombination between Alphasatellite and Betasatellite

**DOI:** 10.3390/v5092116

**Published:** 2013-09-06

**Authors:** Changjun Huang, Yan Xie, Liling Zhao, He Ren, Zhenghe Li

**Affiliations:** Key Laboratory of Molecular Biology of Crop Pathogens and Insects of MOA, Institute of Biotechnology, Zhejiang University, Hangzhou, 310058, China; E-Mails: cjhuang@zju.edu.cn (C.H.); xiey@zju.edu.cn (Y.X.); zhaolilingyunnan@163.com (L.Z.); renhecau@163.com (H.R.)

**Keywords:** begomovirus, recombination, alphasatellite, betasatellite, defective DNA satellite

## Abstract

Monopartite begomoviruses and their associated satellites form unique disease complexes that have emerged as a serious threat to agriculture worldwide. It is well known that frequent recombination contributes to the diversification and evolution of geminiviruses. In this study, we identified a novel defective satellite molecule (RecSat) in association with Tobacco leaf curl Yunnan virus (TbLCYNV) in a naturally infected tobacco plant. Sequence analysis showed that Recsat comprises 754 nucleotides in size and is a chimera involving alphasatellite and betasatellite sequences, containing both betasatellite-conserved region and alphasatellite stem-loop structure. Recombination analysis revealed that RecSat has arisen from three independent recombination events likely involving Tomato yellow leaf curl China betasatellite, Ageratum yellow vein China betasatellite and Tobacco curly shoot alphasatellite. Co-inoculation of RecSat with TbLCYNV induced symptoms indistinguishable from those induced by TbLCYNV alone in *Nicotiana benthamiana*. Southern blot hybridization showed that RecSat could be trans-replicated stably in *N. benthamiana* plants by TbLCYNV, and impaired the accumulation of helper virus and co-inoculated alphasatellite. Our results provide the first evidence for recombination between two distinct types of satellites among geminivirus complex and highlight recombination as a driving force for geminivirus evolution.

## 1. Introduction

Geminiviruses are a group of plant viruses characterized by their geminate shape particles and circular single-stranded DNA (ssDNA) genomes. *Begomovirus*, the largest and the most economically important genus of the *Geminiviridae* family, encompasses viruses that are exclusively transmitted by the whitefly *Bemisia tabaci* and infect only dicotyledonous plants [1]. Over the last 20 years or so, begomoviruses have emerged as serious constraints to the cultivation of a variety of crops in tropical and subtropical regions worldwide [2,3]. Begomoviruses have genomes consisting of either one or two ssDNA components. The two components of bipartite begomoviruses are designated as DNA-A and DNA-B. In contrast, the monopartite begomoviruses lack the component equivalent to DNA-B, with all viral functions encoded by a single component homologous to DNA-A [4]. 

To date, two main types of DNA satellites associated with begomoviruses have been described: betasatellites and alphasatellites. Betasatellites, previously referred to as DNAβ, are satellite molecules associated with monopartite begomoviruses and approximately half the size of the helper virus genome (~1,360 nucleotides in length). Betasatellites modulate disease symptom in most begomovirus/betasatellite complexes and depend on their helper begomoviruses for replication, spreading in plant tissues, encapsidation and insect transmission. The only gene product encoded by betasatellite, βC1, plays an important role in the function of betasatellite. βC1 is a symptom determinant, a suppressor of both transcriptional (TGS) and post-transcriptional gene silencing (PTGS), and can repress plant defenses [5,6,7,8,9,10]. Alphasatellites, formerly known as DNA 1, are also approximately half the size of begomoviral genomes (~1,375 nts) and show a conserved genome organization consisting of a single open reading frame (ORF) coding for a replication initiator protein (Rep) [11,12]. Initially, alphasatellites were found in association with the Old World begomovirus/betasatellite complexes. Recently, some distinct alphasatellites were discovered to be associated with the New World begomoviruses [13,14]. Although alphasatellites were discovered almost 15 years ago, very little is known about the function(s) of these molecules in begomovirus or begomovirus/betasatellite pathogenesis. Alphasatellite and betasatellite share negligible sequence similarity except for an Adenine-rich (A-rich) region, which is hypothesized to be a stuffer sequence that serves to fulfill the size constrain imposed by helper virus-mediated movement or encapsidation [15]. 

Besides the full sized betasatellites and alphasatellites, several defective satellites of approximately 700 nts in length have been identified in begomovirus-infected plants. The first identified subviral agent associated with a geminivirus, referred to as Tomato leaf curl virus satellite (ToLCV-sat), is believed to be a defective betasatellite molecule [15]. ToLCV-sat is a small size (682 nts) molecule without ORF, and is strictly dependent on the helper begomovirus for its replication and encapsidation [15]. Recently, similar begomovirus-associated defective satellites have also been identified in *malvaceous* plants in Cuba and whiteflies in Florida, indicating that this type of satellite molecule is probably common in nature [16,17]. However, it is not known whether this type of vestigial betasatellite plays any role in disease cycle. At least for ToLCV-sat, it affects neither genome accumulation nor symptom expression of its helper virus [15].

Here, a small recombinant satellite, referred to as RecSat, was found in association with the monopartite Tobacco leaf curl Yunnan virus (TbLCYNV). RecSat is 754 nts in size and resulted from recombination between an alphasatellite and betasatellites. Agroinoculation-based infectivity assays demonstrated that RecSat depends on the helper begomovirus for trans-replication and could modulate the accumulation levels of co-inoculated TbLCYNV and alphasatellite.

## 2. Results and Discussion

### 2.1. Results

#### 2.1.1. Identification of a Defective Betasatellite Molecule in Association with TbLCYNV-Infected Tobacco Plant

**Figure 1:**
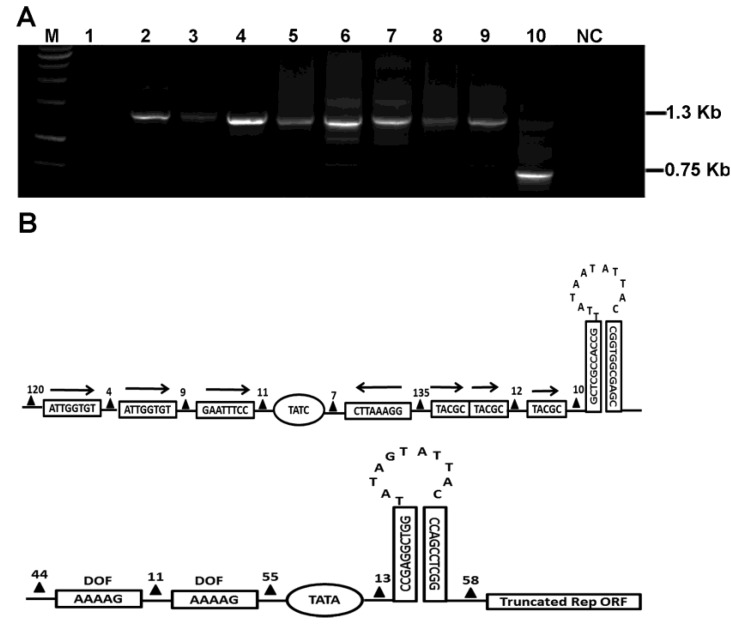
Sequence analysis of RecSat. (**A**) Agarose gel electrophoresis of PCR-amplified products of ten tobacco samples with beta01 and beta02 primers. The DNA products electrophoresed in lane 1–10 wereamplified from sample No. YN51-60, respectively. M denotes size marker lane and NC denotes the healthy control lane. (**B**) Schematic representation of the modular organization of the RecSat betasatellite-like SCR (upper panel) and alphasatellite-like replication origin (lower panel). The arrows represent the position and orientation of the putative iterons. Repeated sequence motifs in RecSat are shown in the box. The triangles with a number on the top indicate the number of spacing nucleotides between elements.

Ten tobacco samples showing characteristic geminivirus-like symptoms were collected from Yunnan, China. Total DNA preparations extracted from these samples were subjected to rolling circle amplification (RCA) followed by conventional PCR. Amplification with the primer pair PA/PB, a begomovirus DNA-A degenerate primer pair [18], yielded a ~500-base pair (bp) product from each sample (data not shown), confirming the begomovirus infection of these samples. To screen for the presence of betasatellite, universal primer pair beta01/beta02 [19] was used in PCR amplification. A fragment of ~1.3 kilobase (kb) was obtained from eight of these 10 samples (Figure 1A). Interestingly, a ~0.75 kb rather than 1.3 kb band was detected from the sample YN60 (Figure 1A). This 0.75 kb fragment was cloned and sequenced, and named RecSat. The presence of alphasatellite was also confirmed by PCR in YN60 sample when amplified with the universal alphasatellite-specific primer pair UN101/UN102 [20]. The amplified DNA-A and alphasatellite DNA fragments from YN60 were cloned and sequenced. Alignment of the determined nucleotide sequence showed that YN60 DNA-A and alphasatellite share the highest nucleotide similarities (~94%) with TbLCYNV and Tobacco curly shoot alphasatellite (TbCSA) (~98%), respectively. All attempts to detect a possible DNA-B component by PCR with DNA-B degenerate primer pairs PCRc1/PBL1v2040 and CR01/CR02 as described [21] from the sample YN60 were unsuccessful, supporting the previous finding that TbLCYNV is a monopartite begomovirus [22]. 

#### 2.1.2. Molecular Characterization of RecSat

Based on the determined RecSat sequence, another pair of abutting primer (RecSatSF and RecSatSR) were designed for PCR amplification, which yielded a specific product of expected size and thus confirmed the circular nature of RecSat (data not shown). Sequence analysis showed that RecSat genome contains 754 nts (GenBank Accession No. KF042891) and has no intact ORF or the A‑rich region, the latter of which is a hallmark of betasatellites and alphasatellites. However, RecSat contains several features of begomovirus-associated betasatellites, including a stem-loop with the conserved nonanucleotide TAATATTAC, and iteron-like motifs located just upstream of the stem‑loop structure (Figure 1B, top panel). Interestingly, another predicted hairpin structure with a loop containing another nonanucleotide TAGTATTAC, which is identical to those of nanoviruses and alphasatellites, was found in the complementary strand of the RecSat. Franking the nonanucleotide of RecSat are some *cis*-elements and regulatory motifs found in the putative promoter sequence of the *Rep* gene of alphasatellite, including TATA-box, DOF motifs (AAAAG), and the 5' truncated *Rep* coding sequence (Figure 1B, bottom panel). The apparent chimeric nature of RecSat suggests that this molecule has been generated as a result of recombination between alphasatellite and betasatellite.

#### 2.1.3. Recombination Analysis of RecSat

**Figure 2:**
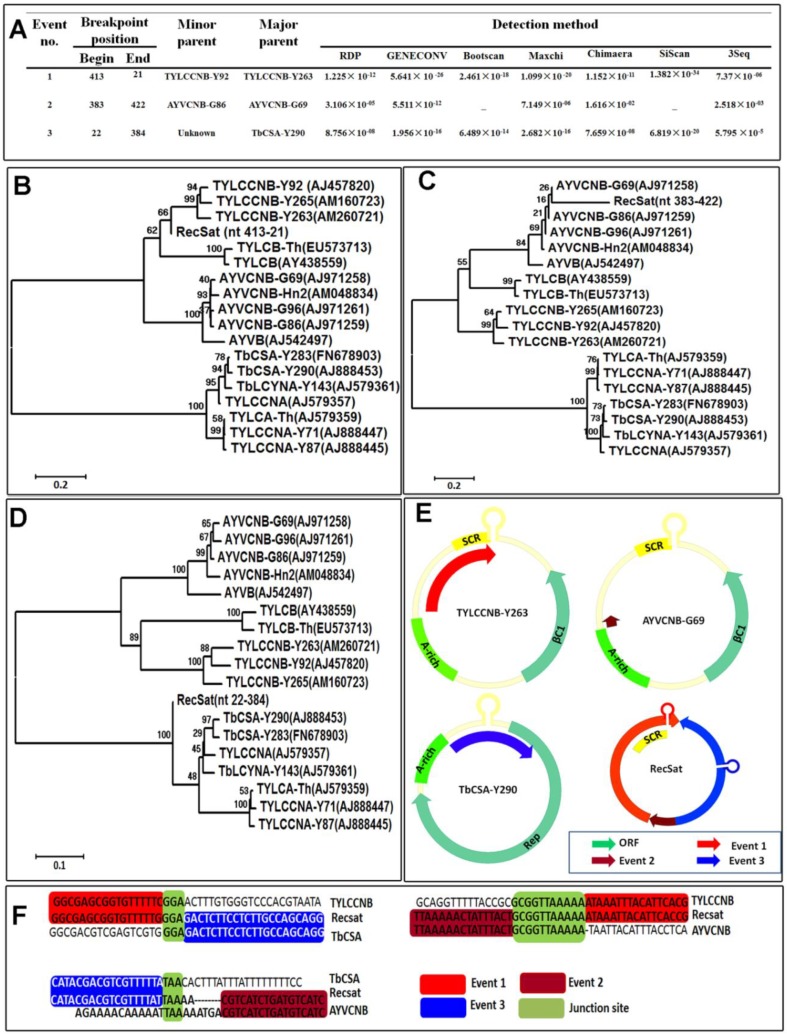
Recombination analyses illustrating the positions of recombination events 1, 2 and 3. The nucleotide position 1 of RecSat has been defined as the A residue immediately downstream of the putative nick site within the SCR (TAATTAC). (**A**) Schematic representation of the three recombination events detected by RDP3 package. Seven different methods available in RDP3 were applied and the obtained confidence values were showed for each recombination event. (**B**–**D**) Neighbor-joining trees were constructed using the RecSat region nt 413–21(**B**), region nt 383–422 (**C**) and region nt 22–384 (**D**), and the most representative betasatellite and alphasatellite isolates from Yunnan, China. Accession numbers are shown in figure and the names of the satellites are as follows: AYVB, Ageratum yellow vein betasatellite; AYVCNB, Ageratum yellow vein China betasatellite; TYLCB, Tomato yellow leaf curl betasatellite; TYLCCNB, Tomato yellow leaf curl China betasatellite; TYLCA, Tomato yellow leaf curl alphasatellite; TbCSA, Tobacco curly shoot alphasatellite; TbLCYNA, Tobacco yellow leaf curl Yunnan alphasatellite. (**E**) Schematic representation of the TYLCCNB-Y263, AYVCNB-G69, TbCSA-Y290 and RecSat. The main genome features such as ORFs, satellite conserved region (SCR) and A-rich region are shown. Sequence regions implicated in recombination event 1, 2 and 3 are displayed in red, dark red and blue, respectively. (**F**) Comparisons of nucleotide sequences of RecSat with TYLCCNB, AYVCNB and TbCSA around junctions. Residues shared by parental sequences at the junction points are shown in green box.

Next, we aligned the RecSat, betasatellite and alphasatellite genome sequences obtained from public sequence databases using ClustalX software (default settings) [23], followed by recombination analysis using the methods (RDP, GENECONV, Bootscan, MaxChi, Chimaera, SiScan, and 3Seq) included in the RDP3 package with default settings. Three potentially significant recombination events were identified with a high degree of confidence by seven recombination detection methods implemented in RDP3 (Figure 2A). The first potential recombinant fragment of 357 nt in size located from nt 421–21 in RecSat (The third adenosine residue within the nonanucleotide TAATTAC derived from betasatellite was defined as the No. 1 nucleotide position). This fragment contains the conserved common region (SCR) of betasatellite, which was likely derived from Tomato yellow leaf curl China betasatellite (TYLCCNB) isolate Y263 (TYLCCNB-Y263). The second potential recombinant region 383–422 shares the highest similarity with Ageratum yellow vein China betasatellite (AYVCNB) isolate G69 (AYVCNB-G69). The third potential recombinant fragment (region 22–384), which contains the alphasatellite conserved stem loop structure and the 5' end of the Rep ORF, can be traced back to TbCSA isolate Y290 (TbCSA-Y290)-like ancestors.

To confirm the phylogenetic relationship, the three relevant regions, divided on the basis of recombination events (event 1: nt 421 to 21, event 2: nt 383 to 422 and event 3: nt 22 to 384), were separately analyzed on neighbor-joining trees with representative betasatellite and alphasatellite isolates. The recombinant RecSat clustered closely with TYLCCNB and AYVCNB sequences, based on event 1 (Figure 2B) and event 2 regions (Figure 2C), respectively, whereas event 3 region clearly clustered with TbCSA sequences (Figure 2D), confirming the occurrence of a triple recombination event.

Further inspection of the recombination breakpoints identified some common sequences between the parental sequences for each recombination event. For example, GGA and TTA were found at the junction site of TYLCCNB/TbCSA, and of TbCSA/AYVCNB, respectively, while a longer stretch of sequence GCGGTTAAAAA was found at the TYLCCNB/AYVCNB junction site (Figure 2F). This suggested that all three recombination events were facilitated by the homologous nucleotide residues of their parental sequences. 

#### 2.1.4. Transreplication of RecSat by TbLCYNV in *Nicotiana benthamiana*

**Figure 3:**
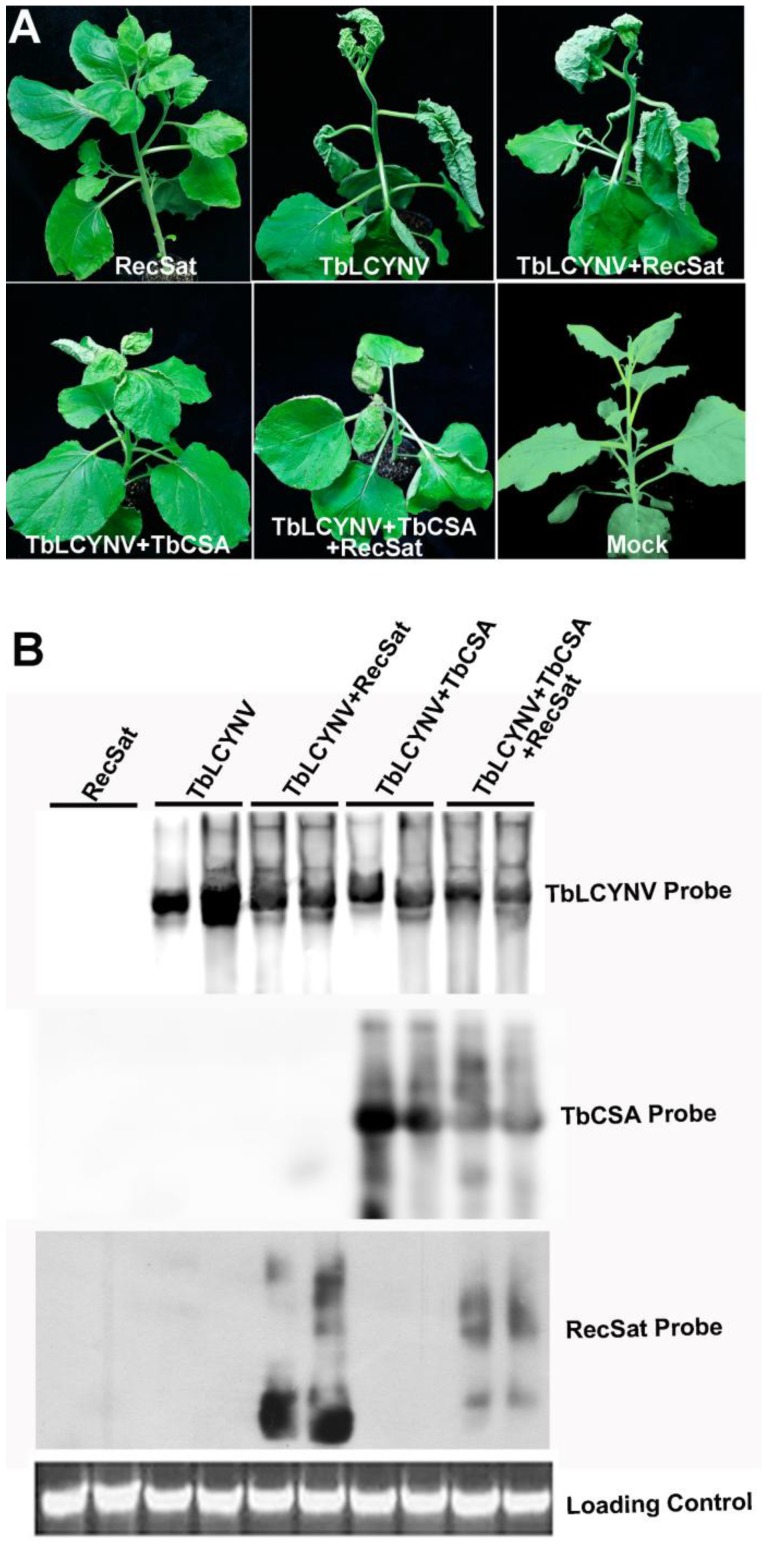
Effect of RecSat on the symptom development and DNA accumulation of helper virus. (**A**) Symptoms induced by TbLCYNV when co-inoculated with either RecSat or TbCSA/RecSat in *Nicotiana benthamiana* at 15 dpi. (**B**) Southern blot analysis of TYLCCNB, TbCSA and RecSat accumulation in *N. bethamiana* plants with specific probes.

To investigate the pathogenicity of this natural recombinant molecule, an infectious clone of RecSat was constructed and agroinoculated into *N. benthamiana* either alone or together with TbLCYNV or TbLCYNV/TbCSA. The infectious clone containing tandem-repeat of the RecSat failed to produce any symptom when agro-inoculated into *N. benthamiana* alone (Figure 3A). Co-inoculation of RecSat and TbLCYNV produced comparable symptom (e.g., severe upward curling of leaves, vein thickening or stunt symptoms) as those induced by TbLCYNV alone in *N. benthamiana* (Figure 3A). In addition, TbLCYNV, TbCSA and RecSat together infected in *N. benthamiana* resulted in mild leaf-curling, vein thickening or stunt symptoms, which are similar to the symptoms observed in the TbLCYNV and TbCSA co-infected *N. benthamiana* plants. These results demonstrated that RecSat had no significant effect on the symptom development of TbLCYNV. 

Southern blot analysis showed that RecSat alone could not systemically spread and accumulate in *N. benthamiana*. When co-inoculated with TbLCYNV, high levels of the RecSat were detected in systemically infected tissue, indicating efficient trans-replication and systemic movement mediated by the helper virus. Comparison of TbLCYNV genomic DNA levels showed that RecSat interfered with helper virus accumulation in co-inoculated plants tissues. When RecSat was co-inoculated with TbLCYNV and TbCSA, attenuations of both TbLCYNV and TbCSA DNA accumulation were also observed. Therefore, these results demonstrated that RecSat could be trans-replicated and stably maintained by TbLCYNV in plants and has a negative effect on the accumulation of TbLCYNV and TbCSA.

### 2.2. Discussion

It is well recognized that genetic recombination plays a major role in the diversification and evolution of geminiviruses [24,25,26]. Recombination has been documented to occur between geminivirus, between betasatellites, alphasatellites and between helper viruses and betasatellites [24,27,28,29,30]. In the present work, we characterized a novel chimeric molecule (RecSat) resulting from recombination between betasatellite and alphasatellite. Using computer program-based recombination analysis, we provided strong evidence that the RecSat genome was generated through three independent recombination events. Inspection of the junction site identified 3–10 common nucleotide residues (e.g., GGA, TAA and GCGGTTAAAAA) shared by parental molecules involved in the recombination events. Such short stretches of common sequences have also been found at the junction site of progenitor geminiviral sequences implicated in other recombination events [28,29]. These results suggest that sequence homology may play an important role in recombination within geminiviruses and their associated satellites. 

The identified “parents” of the recombinant RecSat include TYLCCNB, AYVCNB and TbCSA, all of which are frequently found in association with tobacco-infecting begomoviruses from Yunnan [11,31,32]. Since these “donor” satellites and RecSat have natural hosts in common and prevail in the same geographic location, it is probable that the recombination event may have occurred in co-infected tobacco plants. Alternatively, recombination might have occurred in weeds followed by insect transmission to tobacco plants, since it is well known that weeds are intermediate hosts and reservoirs of begomoviruses and act as “melting pots” that yield new viruses/virus strains by recombination due to their frequently harboring multiple viruses [2]. 

The SCR of betasatellite contains several important *cis*-elements required for trans-replication, including the Rep binding sites and a conserved stem-loop motif that includes the nick site for the initiation of rolling circle replication [33]. All hitherto characterized defective betasatellite molecules retain the SCR, highlighting the importance of this region in the maintenance of betasatellite [15,16,17]. As with defective betasatellites, RecSat also contains the conserved betasatellite stem-loop structure and flanking repeated sequence motifs (Figure 1B) that presumably are involved in recruiting virus‑encoded Rep to initiate replication. Indeed, RecSat accumulated to high levels in the presence of TbLCYNV. Notably, RecSat accumulates at the expense of helper virus replication (Figure 3B). This is reminiscent of defective interfering DNAs, which also appear to decrease helper virus accumulation [20]. It is worth noting that RecSat contains, besides SCR, an alphasatellite-derived replication origin (Figure 1B, Figure 3E), and also interferes with the accumulation of the co-inoculated alphasatellite TbSCA. It is tempting to speculate that alphasatellite alone could mediate the replication of RecSat. Further study is needed to determine whether the alphasatellite-derived replication origin is involved in RecSat replication and interference of the accumulation of both helpers and alphasatellites.

Previous study has showed that only a few isolates of TbLCYNV are associated with betasatellite molecules [21]. Moreover, TbLCYNV alone is able to induce severe symptoms, and co-inoculation with betasatellites does not further intensify the symptoms [22]. Thus, TbLCYNV resembles a true monopartite begomovirus such as ToLCV more than a helper/betasatellite disease complex. The monopartite nature of TbLCYNV may allow defective recombinant molecules as RecSat to be maintained in the absence of functional betasatellite, a scenario reminiscent of ToLCV-sat (15). By contrast, other betasatellite-dependent helper viruses, including Tomato yellow leaf curl China virus, Cotton leaf curl Multan virus and Ageratum yellow vein virus would not prevail in the absence of functional betasatellite. Accordingly, all naturally occurring betasatellite recombinants in association with these helper viruses maintain intact βC1 gene and are able to induce typical symptoms when co‑inoculated with helper viruses [27,28]. Although RecSat appears to have no discernible effect on symptom development in *N. benthamiana* (Figure 3B), its ability to alter helper virus accumulation suggests a biological role as a defective interfering DNA. Further survey of similar molecules in plants and whiteflies will be performed to evaluate their diversity and potential role in disease maintenance.

## 3. Experimental

### 3.1. Sources of Virus Isolates

Ten leaf samples were collected from naturally infected tobacco plants showing begomovirus-like infection symptoms from Yunnan Province, China during the summer of 2003. Total DNAs were extracted from these samples using a CTAB-based method, and rolling-circle amplification (RCA) was performed using a Templiphi^TM^ DNA Amplification Kit (GE Healthcare, Piscataway, NJ, USA) as described [34]. 

### 3.2. PCR Amplification and Cloning of RecSat

Presence of begomovirus in samples was investigated by PCR using RCA product as template and the degenerate primer pair PA/PB as described [34]. In parallel, alphasatellites and betasatellites were analyzed using abutting primers UN101 (5'-AAGCTTGCGACTATTGTATGAAAGAGG-3')/UN102 (5'-AAGCTTCGTCTGTCTTACGAGCTCGCTG-3'), and beta01/beta02 [19] specific to alphasatellites and betasatellites, respectively. The PCR products were purified, and cloned using pGEM-T Easy Vector (Promega, Madison, WI, USA) and sequenced using an automated DNA sequencing system (Model 377; Perkin Elmer, Foster City, CA, USA).

### 3.3. Sequence Analysis

Database searches with the RecSat sequences were carried out by NCBI-BLAST program [35]. Pairwise comparisons were conducted with the ClustalX program using default parameters [23] and phylogenetic inference was performed using MEGA 5 by the neighbor-joining method [36]. Recombination analysis was performed using the methods included in the RDP 3.0 package with default settings [37]. To confirm the recombination results, the relevant fragments from betasatellite or alphasatellite were analyzed on neighbor-joining trees using MEGA 5. 

### 3.4. Construction of RecSat Infectious Clone

To construct the RecSat infectious clone, specific primers RecSatSF (5'-ttacGAGCTCCGGGGAGTTTTTGGAGAGA-3', underlined is an existing *Sal*I restriction site) and RecSatBR (5'-ttatGGATCCCGGGGAGTTTTTGGAGAGA-3', underlined is an existing *Bam*HI restriction site) were designed and used to amplify the full length RecSat genome. The 0.75 kb PCR product was cloned into pGEM-T Easy vector to produce pGEM-1RecSat. After confirmation by sequencing, a *Bam*HI-*Sal*I digested fragment of pGEM-1RecSat was introduced into the binary plant transformation vector pBINPLUS [38] to produce the clone PBINPLUS-1RecSat. Subsequently, using the same strategy, another copy of a full length RecSat genome was amplified with primers RecSatSF and RecSatSR (5'-ttacGAGCTCCGATTAAGGGTCTTCCGGTA-3', underlined is an introduced *Sal*I restriction site) and *Sal*I-digested followed by insertion into the unique *Sal*I restriction site of PBINPLUS-1RecSat to produce clone PBINPLUS-2RecSat, yielding a 2-mer tandem repeat of RecSat. The infectious clones of TbLCYNV (pBINPLUS-Y143) and TbCSA (pBINPLUS-Y35DNA1) were previously constructed in our lab [20,22]. 

### 3.5. Agroinoculation of Plants

The binary vector carrying TbLCYNV, TbCSA and RecSat constructs were introduced individually into *A. tumefaciens* strain EHA105 through electroporation using the GENE PULSER II electroporation system (Bio-Rad Laboratories, Hercules, CA, USA) as instructed by the manufacturer’s manual. The transformed *A. tumefaciens* cultures were incubated individually in a YEP medium containing spectinomycin (50 mg/L) and rifampicin (50 mg/L). The cultures were grown overnight inside a shaker set at 28 °C and 200 rpm. The overnight cultures were pelleted and then resuspended in an induction buffer containing 10 mM MgCl_2_, 10 mM MES, and 150 μM acetosyringone. After 3 h of incubation at room temperature, the *Agrobacterium* cultures were adjusted to OD600 = 0.8 to 1.0, and injected into stem or petioles 4 weeks old *N. benthamiana* plants. For co-inoculation, equal volumes of the separate cultures were mixed prior to inoculation. Inoculated plants were grown in an insect-free chamber and observed daily for the presence of symptoms.

### 3.6. Analysis of Virus and Satellite DNA Accumulation

Nucleic acids were isolated from upper leaves at 30 days post inoculation (dpi) and viral DNA accumulation was assayed by Southern blot hybridization as described [39]. In brief, nucleic acids were fractionated in 1% w/v agarose gel, transferred to Hybond-N^+^ membranes (GE Healthcare) and cross-linked with UV illumination. After alkali denaturation and neutralization, nucleic acid accumulation was detected with a random primed [α-^32^P] dCTP-labeled probe. For analysis of TbLCYNV, a probe containing AC2 and parts of AC1 gene (approximately 710 bp) was amplified from pBINPLUS-Y143 with Y143F (5'-AAGTCTGAGGTTGTAAGCGAGTC-3') and Y143R (5'-CCATCAGTATTGTCATAGAGGGAG-3') primers as described previously [15]. For specific analysis of TbCSA, a probe contains partial TbCSA (nt 500–800 of TbCSA) was amplified from pBINPLUS-Y35DNA1 with TbCSAPF (5'-cggaggaagaattccaggcta-3') and TbCSAPR (5'-aactatattgcagataatcct-3') primers. For specific analysis of RecSat, a probe containing betasatellite homologous sequence (nt 421–721 of RecSat) was amplified from pBINPLUS-2RecSat with RecSatPF (5'-CATTCACCGCATAGGTAAAT-3') and RecSatPR (5'-GCGAGCTAAGCTCCGGCGT-3') primers.

## 4. Conclusions

In summary, we report for the first time the occurrence of recombination between different types of satellites of geminivirus complex in the field. The recombinant satellite interferes with helper virus accumulation, behaving like a defective interfering DNA. Our study highlights the potential of recombination as a driving force for geminivirus diversification.
